
JOURNAL INFORMATION
==============================
NLM Title Abbreviation: J Cardiothorac Surg
Journal ID: J Cardiothorac Surg
NLM Journal ID: 101265113

Journal of Cardiothoracic Surgery

EISSN: 1749-8090
Publisher: BMC

ARTICLE INFORMATION
==============================
PMCID: PMC3870388
DOI: 10.1186/1749-8090-8-S1-O266
Article ID: 1749-8090-8-S1-O266
Article version: 1
Subjects: Oral Presentation

Abstract withdrawn

Publication date: 2013
Electronic publication date: 2013 Sep 11
Volume: 8
Issue: Suppl 1
Supplement: 23rd World Congress of the World Society of Cardio-Thoracic Surgeons: AbstractsPublication of this supplement was funded by the Croatian Thoracic Society.
First page: O266
Last page: O266
Copyright: Copyright © 2013 Popov et al; licensee BioMed Central Ltd.
Copyright year: 2013
Copyright holder: Popov et al; licensee BioMed Central Ltd.
License: This is an Open Access article distributed under the terms of the Creative Commons Attribution License (http://creativecommons.org/licenses/by/2.0), which permits unrestricted use, distribution, and reproduction in any medium, provided the original work is properly cited.
License URL: https://creativecommons.org/licenses/by/2.0/

Conference date: 12-15 September 2013
Conference name: 23rd World Congress of the World Society of Cardio-Thoracic Surgeons
Conference location: Split, Croatia

==============================
*Journal of Cardiothoracic Surgery* 2013, **8**(Suppl 1):O266
http://www.cardiothoracicsurgery.org/content/8/S1/O266

JOURNAL OF CARDIOTHORACIC SURGERY

ORAL PRESENTATION **Open Access**

### Abstract withdrawn

*From* 23rd World Congress of the World Society of Cardio-Thoracic Surgeons
Split, Croatia. 12-15 September 2013

Published: 11 September 2013

doi:10.1186/1749-8090-8-S1-O266
**Cite this article as:** : Abstract withdrawn. *Journal of Cardiothoracic Surgery* 2013 **8**(Suppl 1):O266.

**Submit your next manuscript to BioMed Central and take full advantage of:**

- Convenient online submission
- Thorough peer review
- No space constraints or color figure charges
- Immediate publication on acceptance
- Inclusion in PubMed, CAS, Scopus and Google Scholar
- Research which is freely available for redistribution

Submit your manuscript at
www.biomedcentral.com/submit

BioMed Central
